# A new tool to measure approaches to supervision from the perspective of community health workers: a prospective, longitudinal, validation study in seven countries

**DOI:** 10.1186/s12913-018-3595-7

**Published:** 2018-10-22

**Authors:** Frédérique Vallières, Philip Hyland, Eilish McAuliffe, Ilias Mahmud, Olivia Tulloch, Polly Walker, Miriam Taegtmeyer

**Affiliations:** 1 Centre for Global Health, Trinity College Dublin, 7-9 Leinster Street South, Dublin 2, Ireland; 2 School of Business, National College of Ireland, Mayor Street, IFSC, Dublin 1, Ireland; 3 School of Nursing, Midwifery and Health Systems, University College Dublin, Dublin 4, Ireland; 4 James P Grant School of Public Health, BRAC University, 68 ShahidTajuddin Ahmed Sharani, Mohakhali, Dhaka, 1212 Bangladesh; 5 College of Public Health and Health Informatics, Qassim University, Bukayriah, Qassim Kingdom of Saudi Arabia; 6 Options Consultancy Service, St Magnus House, 3 Lower Thames Street, London, EC3R 6HD UK; 7 Global Centre for Health, HIV and WASH, World Vision International, Victoria Charities Centre, 11 Belgrave Road, London, SW1V 1RB UK; 8 Department of International Public Health, Liverpool School of Tropical Medicine, Pembroke Place, Liverpool, L3 5QA UK

**Keywords:** Community health workers, Supervision, Perceived supervision scale, Motivational outcomes, Scale validation

## Abstract

**Background:**

The global scale-up of community health workers (CHWs) depends on supportive management and supervision of this expanding cadre. Existing tools fail to incorporate the perspective of the CHW (i.e. perceived supervision) in terms of supportive experiences with their supervisor. Aligned to the WHO’s strategy on human resources for health, we developed and validated a simple tool to measure perceived supervision across seven low and middle-income countries.

**Methods:**

Phase 1 was carried out with 327 CHWs in Sierra Leone. Twelve questions, informed by the extant literature on health worker supervision, were reduced to six questions using confirmatory factor analysis. Phase 2 employed structural equation modelling with 741 CHWs in six countries (Bangladesh, Ethiopia, Indonesia, Kenya, Malawi, Mozambique), to assess the factorial validity, predictive validity, and internal reliability of the questions at three time-points, over 8-months.

**Results:**

We developed a robust, 6-item measure of perceived supervision (PSS), capturing regular contact, two-way communication, and joint problem-solving elements as being critical from the perspective of CHWs. When assessed across the six countries, over time, the PSS was also found to have good validity and internal reliability. PSS scores at baseline positively and significantly predicted a range of performance-related outcomes at follow-up.

**Conclusion:**

The PSS is the first validated tool that measures supervisory experience from the perspective of CHWs and is applicable across multiple, culturally-distinct global health contexts with a wide range of CHW typologies. Simple, quick to administer, and freely available in 11 languages, the PSS could assist practitioners in the management of community health programmes.

**Electronic supplementary material:**

The online version of this article (10.1186/s12913-018-3595-7) contains supplementary material, which is available to authorized users.

## Background

The important role of lower-cadre health workers in achieving Universal Health Coverage (UHC) is widely recognised, with community health workers (CHWs) frequently cited as a cost-effective, critical resource for the efficient delivery of primary care in low- and middle-income contexts (LMICs) [1, 2]. Unfortunately, scaling up and sustaining CHWs programme, as envisioned at Alma-Ata, has been challenging, with wide variations in the availability, coordination, support and management of community health worker programmes [3]. Accordingly, the most recent *Global strategy on human resources for health: Workforce 2030* [4] published by the World Health Organization (WHO) reiterates the need to harness the potential of community-based health workers. Specifically, the strategy calls for a global effort to integrate CHWs into national health-care systems as a means to improve their working conditions, capacity, and motivation [4].

More recently, the WHO have called for rigorous scientific research in the area of community health workers to pay more attention to cross-cutting factors, such as management and supervision, that enable community-based health worker performance [5]. Decades of research on CHW initiatives to date have suggested several cross-cutting factors that contribute to the success of CHW programmes [6]. Among these, supportive supervision consistently emerges as a key factor in determining CHW performance, motivation, and retention [7].

In contrast to more ‘traditional’ methods of supervision, which are frequently characterised by performance audits, inspections, use of checklists, and controlling and authoritarian attitudes [7–10], supportive supervision favours shared performance goals, mentoring, and two-way communication [11]. Whereas traditional approaches are frequently criticised for their failure to enhance health worker motivation [12–14], supportive approaches to supervision have been shown to increase the impact of CHW programmes as well as the productivity, motivation and job satisfaction of CHWs [7, 15–17]. Moreover, CHWs themselves express clear preferences for supportive approaches that are responsive to the realities of the challenges they face in programme implementation [14, 18].

In addition to supportive approaches to supervision, CHW programmes often advocate for regular supervision of CHWs. Research suggests however that regular interaction with one’s supervisor is insufficient. When compared to colleagues who had recently been supervised *and* felt supported by their supervisor, health workers who had recently been supervised, but did *not* feel supported, were found to be less productive [15]. This suggests that not only are health worker’s perceptions of the supervisory relationship significant, but that perceptions of the supportive nature of this relationship is likely a more important predictor of work-related outcomes than frequency alone. This view is consistent with well-established theories within the work psychology literature, which state that subjective, cognitive appraisals of supervision are critical factors in the prediction of a range of work performance-related factors (e.g., motivation, commitment, job satisfaction) [19].

While existing tools measure the supervision of CHWs (i.e. the “CHW Assessment and Improvement Matrix” [20]) by assessing the frequency of supervision and training of supervisors, these measures crucially ignore CHW perceptions of the supervisory process and their impact on work-performance-related factors. Moreover, such tools are lengthy, time-intensive, and require substantial programmatic input and resources; all of which are at a premium within human resource for health programming in LMICs. The need exists to develop a feasible, valid, and reliable measure of perceived supervision that both recognises the experience of supervision from the perspective of the individual health worker and that allows the CHW voice to be heard.

The current study aimed to develop and psychometrically validate a new, simple measure of perceived supervision (the *Perceived Supervision Scale* (PSS)) that could be used across multiple global health contexts. To maximise the utility of the PSS in LMICs we sought to construct an easily-translatable measure, comprised of a limited number of items that can be quickly and easily administered and scored; an approach that should increase the likelihood of cross-cultural validity and subsequent use.

The development and validation of the PSS included two research phases. Phase 1, conducted in Sierra Leone, was exploratory and sought to determine the most appropriate indicators of perceived supervision from an initial pool of test items. In other words, we sought to determine which items, when included in a questionnaire, measured perceived supervision among CHWs. Phase 2, conducted across six LMICs and over a period of 8 months, sought to provide a comprehensive assessment of the psychometric properties of the PSS. Specifically, this phase assessed the predictive validity, factorial validity, cross-cultural and temporal stability of the factor structure, and the internal reliability of the PSS over time and across multiple cultural contexts. In other words, we sought to determine whether the questionnaire, as developed in the Sierra Leonean context also measured perceived supervision among CHWs across six other contexts, and whether measures of perceived supervision using the PSS at baseline, predicted a number of related human resource for health outcomes 8-months later. Additionally, we assessed whether the total score on the PSS could be used by implementers in the management and monitoring of CHW programmes.

## Methods

### Participants and procedures

Phase 1 was conducted in Bonthe District, Sierra Leone among a convenience sample of 327 CHWs, representing 98% of the CHWs active in the four chiefdoms of Jong, Imperi, Sogbeni, and Kpanda Kemoh. Data collection took place over 3 weeks in May 2012 as part of a longitudinal cohort study of CHWs participating in World Vision Ireland’s Access to Infant and Maternal Health (AIM-Health) programme. Phase 2 recruited a convenience sample of 741 CHWs from an additional six countries (Bangladesh, Ethiopia, Kenya, Indonesia, Malawi and Mozambique) all of whom were assessed across three time periods (baseline [T0], 4 months [T1], and 8 months [T2]). CHWs were recruited in consultation with either national ministries of health (Bangladesh, Malawi, Mozambique, Kenya), regional (Ethiopia) or district-level health management teams (Indonesia), and based on the presence of a functioning CHW programme in these districts. Data collection took place between October 2014 and May 2015 as part of the REACHOUT research consortium (www.reachoutconsortium.org). Demographic information for all participants is reported in Table 1.

**Table 1:** Summary of CHW demographics and sampling methods employed across all seven study locations

	Sierra Leone	Bangladesh	Ethiopia	Indonesia	Kenya	Malawi	Mozambique
Cadre	CHW	CTC	HEW	Village midwives	CHW/CHEW	HSA	APE
Sampling method	Convenience	Convenience	Convenience	Convenience	Convenience	Random	Convenience
Language^a^	Krio, Mende, English	Bangla	Amharic	Basha-Indonesia	English, Swahili, Kamba	Chichewa	Portuguese, Ronga, Changane
Sample size	327	119	108	230	51	124	78
Sex (Female %)	54.7%	75.6%	100%	97.0%	76.5%	38.7%	61.5%
Completed Secondary Education	28.9%	12.3%	99.1%	28.3%	34.1%	79.5%	0%

*CHW* Community health worker, *CTC* Close to Community health service providers, *HEW* Health extension worker, *CHEW* Community health extension worker, *HAS* Health surveillance assistants, *APE* Agentes polivalentes elementares, *SD* Standard deviation

^a^In the case of oral languages (i.e. Mende), the questionnaire was ultimately left in English and administered by an enumerator in the language most familiar to the participants

### Development of the initial tool

The 12 items of the PSS were initially constructed to capture aspects of supervision described in the literature [21, 22]. Items are scored using a 5-point Likert scale anchored by “strongly disagree” (1) and “strongly agree” (5). Items were designed to capture key components of supervision, as identified from the literature, including perceptions of regular contact (My supervisor meets with me regularly) and strong two-way communication (My supervisor meets with me regularly to discuss problems and solutions; My supervisor takes into consideration my views and ideas; and My supervisor is a good communicator). These items were first translated in Phase 1 into Krio, Sierra Leone’s lingua franca. During Phase 2, the refined version of the PSS was further translated into seven additional languages (Bangla, Kiswahili, Kamba, Bahasa-Indonesia, Chichewa, Portuguese, and Amharic). Translated forms of the PSS are available for free download at www.perceivedsupervisionscale.com. All versions were piloted, revised, back-translated, and compared to the original English version prior to being administered by trained enumerators. In the case of illiterate CHWs, the PSS was administered with the help of an enumerator. In the case of literate CHWs, the PSS was completed directly by the CHW. In both phases, enumerators were trained to administer the PSS in the local languages and English.

In Phase 2, work-performance related factors were also assessed over time. Adapted from Mbindyo et al. [23], the *Motivational Outcome Scale* is a 12 item, self-report measure of work-performance related constructs: community commitment (2 items, *α* = .64), organizational commitment (2 items, *α* = .44), job satisfaction (4 items, *α* = .73), and work conscientiousness (4 items, *α* = .73). Each item was assessed using a 5-point Likert Scale, anchored by “strongly disagree” (1) and “strongly agree” (5). Among the current sample, the scale possessed satisfactory internal reliability.

### Analysis

During Phase 1, the initial pool of 12 PSS items were assessed using confirmatory factor analysis (CFA) to develop a short, unidimensional measure of perceived supervision (see Additional file 1: Table S2). CFA is a statistical technique that tests whether items in a questionnaire effectively measure a theoretical construct, or *latent construct*, that is itself not directly observable (i.e. perceived supervision) [24]. As Phase I was more exploratory in nature, we did not expect all 12 items to measure perceived supervision in a consistent and robust manner. To determine which of these 12 items should be retained as the best measures of perceived supervision, we set an a priori criterion for item retention whereby only items with factor loadings^1^ >.55 (equalling 30% of variance explained by the latent variable) were retained [25]. In addition to consulting factor loadings, we also consulted modification indices produced in Mplus (Version 7.4). Modification indices provided suggestions of additional items that could be removed to improve model fit (i.e. items with covarying residuals) [26].

Phase 2 also used CFA procedures to determine the factorial validity of the PSS. In addition, structural equation modelling (SEM) methods were used to assess whether perceived supervision scores, as measured by the PSS at baseline (Time 0), predicted the four criterion variables of the Motivational Outcomes Scale at endline (Time 2), controlling for sex and educational status. Here, SEM was chosen to assess the predictive validity of the PSS as it allows for all effects in the model to be estimated simultaneously. In other words, SEM methods were used to test whether the administration of the PSS scale at earlier stages of CHW programmes predicted a range of meaningful human resource for health-related outcomes throughout later stages of a CHW programme, whereby job satisfaction, organizational commitment, community commitment, and work conscientiousness were measured as known determinants of CHW programme success. The internal reliability of the PSS was assessed using composite reliability analysis [27], and descriptive statistics were calculated for each country and at each assessment period.

Analyses were conducted in Mplus 7.4 [28] using the mean and variance-adjusted weighted least squares (WLSMV) estimator. The WLSMV estimator provides accurate parameter estimates, standard errors, and test-statistics when ordinal indicators are used [29]. Missing data was managed using the default pairwise present analysis method. Standard recommendations for assessing the fit of the CFA and SEM models were followed [30] whereby a non-significant chi-square (χ2) result indicates good model fit; Comparative Fit Index (CFI) and Tucker Lewis Index (TLI) values >.90 indicate good fit; Root-Mean-Square Error of Approximation (RMSEA) with 90% confidence interval (RMSEA 90% CI) values <.08 reflect good fit; and values <1.0 for the Weighted Root Mean Square Residual (WRMR) indicate good model fit. In other words, models that met these criteria were seen to be a ‘good’ representation of perceived supervision.

## Results

### Phase 1: development of the perceived supervision scale

The fit of the unidimensional, 12-item model to the sample data was poor (χ^2^ = 355.417, df = 54, *p* < .001; CFI = .757; TLI = .703; RMSEA [90% CI] = .131 [.119–.145]; WRMR = 1.739). Inspection of the model parameters indicated that six items failed to reach the a priori criterion of factor loadings >.55 on the Perceived Supervision factor (Additional file 1: Table S2). The unidimensional model was subsequently re-estimated based on the remaining six items and model fit was acceptable (χ^2^ = 43.952, df = 9, *p* < .001; CFI = .961; TLI = .934; RMSEA [90% CI] = .110 [.079–.143]; WRMR = .910). The factor loadings for the six items were all positive, statistically significant, and of a robust magnitude.

### Phase 2: validity of the perceived supervision scale

Table 2 reports the CFA results for the six-item, unidimensional model of the PSS across six nations, and at three assessment periods. In most cases the χ2 values were statistically significant and the RMSEA values were above the suggested cut-off point of .08. However, rejection of the models based on these indices is not warranted given the tendency for the χ2 to generate Type 1 errors, and the RMSEA to generate Type 2 errors in models with few degrees of freedom [31]. Contrastingly, the CFI, TLI, and WRMR results provided consistent support for the factorial validity of the PSS. In all 17 assessments, the CFI, TLI, and WRMR results satisfied the criteria for excellent model fit. Overall, the CFA results provide support for the validity of a unidimensional structure of the PSS that is stable over time, and cross-culturally consistent.

**Table 2:** Model fit statistics for the unidimensional model of the Perceived Supervision Scale (PSS)

Country	*χ* ^*2*^	*df*	*P*	CFI	TLI	RMSEA (90% CI)	WRMR	Number
Time 0								
Bangladesh	40.781	9	.000	.953	.922	.194 (.136–.256)	.816	94
Ethiopia	14.130	9	.118	.997	.995	.073 (.000–.142)	.398	107
Indonesia	17.324	9	.044	.964	.940	.097 (.016–.165)	.557	99
Kenya	21.080	9	.012	.995	.991	.162 (.072–.253)	.563	51
Malawi	31.353	9	.000	.982	.971	.142 (.090–.197)	.612	124
Mozambique	21.935	9	.009	.987	.979	.139 (.066–.215)	.616	74
Time 1								
Bangladesh	17.435	9	.042	.992	.987	.094 (.017–.160)	.453	106
Ethiopia	37.267	9	.000	.984	.973	.175 (.119–.235)	.741	103
Indonesia	30.819	9	.000	.946	.910	.156 (.098–.218)	.790	100
Kenya	–	–	–	–	–	–	–	–
Malawi	24.887	9	.003	.977	.962	.128 (.069–.189)	.626	108
Mozambique	39.304	9	.000	.956	.927	.210 (.146–.280)	.811	76
Time 2								
Bangladesh	18.183	9	.033	.997	.995	.112 (.030–.186)	.540	82
Ethiopia	16.836	9	.051	.956	.927	.091 (.000–.158)	.636	105
Indonesia^a^	32.313	8	.000	.959	.924	.174 (.114–.239)	.683	100
Kenya	31.762	9	.000	.990	.983	.223 (.142–.309)	.746	51
Malawi	59.142	9	.000	.944	.907	.212 (.162–.265)	.873	124
Mozambique	34.507	9	.000	.947	.912	.203 (.134–.276)	.793	69

^a^A correlated residual variance was added between PSS4 and PSS6 to achieve acceptable model fit

The standardised factor loadings for the PSS across each nation, at each assessment, are reported in Additional file 1: Table S3. Factor loadings at T0 were all positive, significant (*p* < .001), and robust, with mean factor loadings ranging from .68 (Indonesia) to .92 (Kenya). Similarly, at T1 all factor loadings were positive, significant (*p* < .001), and robust, with mean factor loadings ranging from .74 (Indonesia) to .83 (Ethiopia). At T2, there was greater variability in the performance of the model parameters. Within the Indonesian sample it was necessary to add a residual covariance between two items with the lowest factor loadings (PSS4 and PSS6: factor loadings <.50) to achieve acceptable model fit. Additionally, within the Ethiopian sample two items possessed weak factor loadings (PSS2 = .11 and PSS4 = .22). Nonetheless, mean factor loadings were generally robust, ranging from .50 (Ethiopia) to .91 (Bangladesh).

Given the stability of the unidimensional structure of the PSS across nations, and time, all PSS data at T0 was merged. Model fit of this consolidated data was satisfactory (*N* = 710; χ^2^ = 138.936, df = 9, *p* < .001; CFI = .987; TLI = .979; RMSEA [90% CI] = .143 [.122–.164]; WRMR = .864), and therefore used to assess predictive validity.^2^

### Predictive validity of the perceived supervision scale

A PSS latent variable modelled at T0 was used to predict the summed scores of four criterion variables (job satisfaction, organizational commitment, community commitment, and work conscientiousness) measured 8 months later (T2), controlling for sex and educational status. The fit of the model to the data was excellent (χ^2^ = 91.276, df = 41, *p* < .001; CFI = .991; TLI = .986; RMSEA [90% CI] = .045 [.033–.058]; WRMR = .847). As detailed in Table 3, the model explained between 5.8 and 16.4% of variance in each of the criterion variables, and perceived supervision positively predicted all variables (β values ranged from .16 to .30).

**Table 3:** Predictive effects of the Perceived Supervision Scale at T0 on four criterion variables at T2 (*N* = 602)

	Job Satisfaction		Organizational Commitment		Community Commitment		Work Conscientiousness	
	β	SE	β	SE	β	SE	β	SE
Sex^a^	.12*	.05	.07	.04	.06	.05	.08	.04
Education^b^	.12*	.05	.17***	.05	.13**	.05	.13**	.05
Perceived Supervision	.25***	.04	.16**	.05	.29***	.04	.30***	.04
R^2^	.09***		.06**		.11***		.16***	

^a^Sex is coded 0 = Females, 1 = Males; ^b^ Education is coded 0 = Did not complete secondary schooling, 1 = Completed secondary schooling; β = Standardized beta values; SE = Standard errors for β; R^2^ = percentage of unique variance explained in each criterion variable; *p < .05, **p < .01, ***p < .001

### Internal reliability and descriptive statistics for the PSS

Composite reliability analyses indicated that the PSS possesses satisfactory internal reliability (Additional file 1: Table S3), indicating that the six items were internally consistent and serve as accurate measures of perceived supervision. In every national context, and at each assessment period, the reliabilities ranged from .68 to .97. Descriptive statistics for the PSS across all nations, at each assessment period, are presented in Table 4.

**Table 4:** Descriptive statistics for the Perceived Supervision Scale

	Mean	95% Confidence Intervals of the Mean	Median	Standard Deviation	Range
Time 0					
Bangladesh	26.90	[26.25, 27.56]	27	3.68	13–30
Ethiopia	26.21	[25.44, 27.07]	28	4.47	11–30
Indonesia	23.16	[22.74, 23.59]	24	2.06	15–27
Kenya	22.43	[20.68, 24.18]	24	6.09	7–30
Malawi	23.55	[22.76, 24.34]	24	4.44	11–30
Mozambique	24.65	[23.82, 25.48]	24	3.57	10–30
Time 1					
Bangladesh	27.03	[26.30, 27.76]	29	3.79	10–30
Ethiopia	23.70	[22.73, 24.67]	24	4.94	9–30
Indonesia	23.85	[23.35, 24.35]	24	2.48	16–30
Kenya^a^	–	–	–	–	–
Malawi	24.01	[23.21, 24.50]	24.5	4.20	9–30
Mozambique	25.22	[24.54, 25.91]	25	3.00	16–30
Time 2					
Bangladesh	26.69	[25.67, 27.70]	28	4.56	6–30
Ethiopia	24.18	[23.51, 24.85]	24	3.45	14–30
Indonesia	23.40	[22.93, 23.97]	24	2.60	12–30
Kenya	23.45	[21.67, 25.23]	24	6.33	6–30
Malawi	23.83	[23.09, 24.57]	24	4.16	11–30
Mozambique	25.10	[24.39, 25.82]	25	2.97	16–30

^a^No data available at T1 for Kenya

## Discussion

The Perceived Supervision Scale is the first validated tool developed for collecting CHW perceptions of their supervision. The tool is brief, robust and can be applied across multiple, culturally-distinct global health contexts with a wide range of CHW typologies. Despite its recognised importance of supervision in CHW programming, supervision is often one of the weakest and most difficult elements of CHW programming to implement consistently [9, 32]. The factor structure of the PSS allows researchers and implementers to calculate a sum score of perceived supervision within CHW programming. Specifically, the total PSS score allows for a greater understanding the nature of a positive supervisory relationship. Furthermore, it grants the ability to managers to detect problematic supervisory interactions, prompt the introduction of stronger training programmes, and where necessary, the reorganisation of supervisory arrangements, contributing to the sustainability of CHW programmes. The ability for CHW programme managers to monitor the interpersonal supervisory relationships of CHWs could help prevent deleterious work performance outcomes associated with high staff turnover and loss of worker motivation [7, 33]. The development of the PSS therefore represents a valuable contribution to global efforts to address human resource for health shortages and towards achieving UHC. Furthermore, the development of the PSS contributes towards addressing more recent calls for rigorous approaches towards scale development for human resource for health programming [34].

Phase 1 served to derive the most appropriate indicators of perceived supervision. From an initial pool of 12 item statements, developed from the extant literature on CHW supervision, six items were retained. Consistent with previous literature, the items retained as part of the final PSS, reflect the importance of *both* supportive and regular aspects of supervision. Interestingly, those items associated with more traditional forms of supervision (i.e. controlling or negative interactions), were least reflective of the nature of perceived supervision among this sample of CHWs. This suggests that CHWs in Sierra Leone perceived the supervision process as a generally positive, supportive, and regular experience. The items retained as part of the supportive supervision factor offer additional insight into what content or skills should be emphasised or included as part of supervision training programmes. More specifically, the items retained in the PSS are consistent with evidence that a supportive supervisor should: meet regularly with CHWs, offer opportunities for knowledge sharing and refresher training [33], recognise and appreciate the work and efforts of a CHW, take into account the views and ideas of CHWs, and communicate effectively with the CHW [11].

As it was possible that the observed findings from Phase 1 reflected the idiosyncratic responses of the Sierra Leonean CHWs, it was imperative to assess the replicability of these findings in alternate contexts. Phase 2 confirmed the PSS’s unidimensional structure across multiple samples of CHWs from different contexts, cadres, cultures, and demographics. Additionally, the factorial validity of the PSS was evidenced across time, with the scale exhibiting stable psychometric properties (reliability and validity) over a period of 8 months. Furthermore, the PSS positively predicted a range of work-performance related indicators 8 months later including job satisfaction, work conscientiousness, community commitment, and organizational commitment, while controlling for sex and education. These results indicate that CHWs who perceive greater levels of supervision (i.e. supportive) report greater job satisfaction, work conscientiousness and higher levels of both community and organizational commitment over time. Administering the PSS during early stages of programme implementation, or when used regularly as a monitoring tool, may therefore help managers to adapt supervision approaches before they negatively impact on other organizational factors in the long-term. Although such findings are important, future research should extend upon these findings and assess the effectiveness of the PSS to also predict objective outcomes of CHW performance and community health outcomes.

The current study has several limitations that should be recognised. The selection of the six PSS items was drawn from a sample of CHWs in Sierra Leone, and although the latent structure of these items was confirmed cross-culturally, it is possible that had the scale refinement process been conducted in a different setting, a different set of indicators may have been retained. It is important to note that the PSS is not presented as a comprehensive measure of perceived supervision, but rather a brief measure of the construct that possesses high utility across global health contexts. Second, the country-specific CFA models generated during Phase 2 of the study were carried out using relatively small sample sizes. Although not ideal for latent variable modelling, the small number of indicators in the PSS render this a minor limitation [35]. Third, it is worth noting that a residual covariance was added between two items in one (Indonesia, time 2) of 17 assessments of model fit. Finally, while the PSS has been validated among CHWs across a range of LMIC contexts, it is necessary to determine the reliability and validity of PSS among more highly skilled cadres of health workers globally.

## Conclusion

In comparison to current tools [20] that focus on capturing the frequency and regularity of supervision, the PSS allows for the subjective measurement for supervision as a predictor of future CHW satisfaction, engagement, and commitment. Simple and quick to administer, and currently available in nine languages, the validated PSS has the potential to contribute towards a more accurate understanding of CHW’s perspectives of supervision, as a critical determinant of successful CHW programmes across a wide range of contexts.

## Additional file

Additional file 1: **Table S1.** Summary of Ethical Approvals. **Table S2.** Pre- and post-refinement standardised factor loadings and standard errors for the PSS during Phase I. **Table S3.** Standardized factor loadings (standard errors) for the Perceived Supervision Scale (PSS) and Composite Reliability (CR) results. (PDF 96 kb)

## Footnotes

1 Depicted as λ_pre_ in Additional file 1: Table S2, factor loadings indicate what proportion of the variance in each item on the questionnaire can be explained by the underlying latent construct.

2 A unidimensional model indicates that the PSS should be scored by summing questions PSS1-PSS6 to produce a total PSS score.
