# Supplementary material for: A new tool to measure approaches to supervision from the perspective of community health workers: a prospective, longitudinal, validation study in seven countries
Source: BMC Health Serv Res. 2018 Oct 22;18:806. doi: 10.1186/s12913-018-3595-7 (PMC6196473; doi:10.1186/s12913-018-3595-7)
Supplement: Supplementary file 1 — Table S1. Summary of Ethical Approvals. Table S2. Pre- and post-refinement standardised factor loadings and standard errors for the PSS during Phase I. Table S3. Standardized factor loadings (standard errors) for the Perceived Supervision Scale (PSS) and Composite Reliability (CR) results. (PDF 96 kb) [file 12913_2018_3595_MOESM1_ESM.pdf]

Table S1. Summary of Ethical Approvals

Ethical Review Committee	Country
**Phase I**	
Health Policy and Management/Centre for Global Health Ethical Review Committee, Trinity College Dublin	Ireland
Connaught Hospital, Freetown	Sierra Leone
**Phase II**	
Liverpool School of Tropical Medicine Research Ethics Committee	UK
James P Grant School of Public Health, BRAC University Ethical Review Committee in Bangladesh	Bangladesh
Kenya Medical Research Institute Ethics Review Committee	Kenya
Eijkman Institute Research Ethics Commission in Indonesia	Indonesia
National Health Sciences Research Committee, Ministry of Health Malawi	Malawi
The Research and Technology Transfer Support Process at the Southern Nations, Nationalities and Peoples Regional Health Bureau	Ethiopia
ComitéInstitucional de BioeticaemSaude da Faculdade de Medicina, Hospital Central de Maputo in Mozambique	Mozambique

Table S2. Pre- and post-refinement standardised factor loadings and standard errors for the PSS during Phase I.

Item	λ_{pre}	SE_{pre}	λ_{post}	SE_{post}
My supervisor is unhelpful (R)	-.19	.06	--	--
My supervisor is controlling (R)	.01*	.06	--	--
My supervisor likes to remind me of the rules to follow (R)	.39	.05	--	--
My supervisor meets with me regularly(PSS1)	.57	.05	.51	.05
My supervisor knows when my next supervision meeting will take place	.49	.05	--	--
My supervisor does not give me personal feedback (R)	.17	.05	--	--
My supervisor makes me uncomfortable or embarrassed by discussing my work in front of others (R)	-.01*	.06	--	--
My supervisor appreciates me (PSS2)	.62	.05	.60	.05
My supervisor meets with me regularly to discuss problems and solutions(PSS3)	.57	.05	.53	.05
My supervisor takes into consideration my views and ideas (PSS4)	.75	.03	.77	.03
My supervisor is a good communicator (PSS5)	.76	.03	.77	.03
My supervisor helps me to update my knowledge (PSS6)	.61	.05	.65	.05

Note: n = 323*;* (R) = negatively worded items; *factor loadings are not statistically significant ($p > .05$)

Table S3. Standardized factor loadings (standard errors) for the Perceived Supervision Scale (PSS) and Composite Reliability (CR) results.

	PSS1	PSS2	PSS3	PSS4	PSS5	PSS6	Mean Factor Loading	CR
				**Time 0**				
Bangladesh	.71	.68	.92	.73	.93	.71	.78	.91
	(.07)	(.05)	(.04)	(.06)	(.04)	(.07)		
Ethiopia	.75	.80	.95	.88	.90	.84	.85	.94
	(.05)	(.04)	(.03)	(.04)	(.03)	(.04)		
Indonesia	.49	.89	.49	.46	.82	.93	.68	.85
	(.09)	(.08)	(.11)	(.09)	(.08)	(.06)		
Kenya	.83	.92	.96	.91	.98	.91	.92	.97
	(.04)	(.03)	(.02)	(.03)	(.02)	(.02)		
Malawi	.66	.64	.78	.85	.84	.85	.77	.90
	(.05)	(.05)	(.04)	(.03)	(.04)	(.04)		
Mozambique	.61	.87	.89	.91	.97	.77	.84	.94
	(.06)	(.05)	(.03)	(.05)	(.04)	(.06)		
				**Time 1**				
Bangladesh	.60	.79	.93	.75	.94	.88	.82	.93
	(.06)	(.06)	(.03)	(.05)	(.03)	(.04)		
Ethiopia	.68	.80	.94	.95	.82	.77	.83	.93
	(.05)	(.04)	(.02)	(.02)	(.04)	(.04)		
Indonesia	.67	.62	.70	.80	.74	.88	.74	.90
	(.08)	(.07)	(.07)	(.06)	(.07)	(.05)		
Kenya	--	--	--	--	--	--	--	
Malawi	.63	.70	.78	.80	.91	.82	.77	.90
	(.06)	(.06)	(.05)	(.05)	(.05)	(.05)		

Mozambique	.60	.83	.83	.62	.96	.73	.76	.90
	(.07)	(.07)	(.05)	(.05)	(.03)	(.07)		
				**Time 2**				
Bangladesh	.85	.91	.86	.89	1.00	.95	.91	.97
	(.03)	(.04)	(.04)	(.04)	(.03)	(.03)		
Ethiopia	.50	.11	.61	.22	.73	.80	.50	.68
	(.10)	(.11)*	(.08)	(.10)^	(.07)	(.08)		
Indonesia	.68	.54	.96	.38	.95	.43	.66	.83
	(.06)	(.05)	(.04)	(.07)	(.04)	(.06)		
Kenya	.77	.96	.83	.93	.85	.97	.89	.96
	(.05)	(.02)	(.05)	(.03)	(.04)	(.01)		
Malawi	.51	.51	.78	.81	.84	.87	.72	.87
	(.06)	(.05)	(.05)	(.04)	(.04)	(.03)		
Mozambique	.60	.82	.79	.53	.91	.66	.72	.87
	(.07)	(.08)	(.05)	(.06)	(.04)	(.08)		

Note: All standardized factor loadings are significant ($p < .001$) except * ($p > .05$) and ^ ($p < .05$).
